# Atorvastatin accelerates hematoma absorption and ameliorates cognitive impairment in patients with chronic subdural hematoma complicated by cognitive dysfunction: a retrospective analysis

**DOI:** 10.3389/fphar.2026.1816058

**Published:** 2026-07-09

**Authors:** Sen He, Li Zhang, Fang Xue, Wenyan Zhang, Fei Xie

**Affiliations:** Department of Neurosurgery, West China Hospital of Sichuan University-Ziyang Hospital and Ziyang Central Hospital, Ziyang, Sichuan, China

**Keywords:** atorvastatin, chronic subdural hematoma, cognitive impairment, conservative treatment, hematoma absorption, neuroprotection

## Abstract

Chronic subdural hematoma (CSDH) is a prevalent neurosurgical disorder in the elderly, often accompanied by cognitive impairment that severely compromises prognosis. Atorvastatin promotes hematoma absorption, exerts anti-inflammatory and neuroprotective effects, but its efficacy, cognitive improvement and safety in CSDH patients with cognitive impairment remain unclear. This study aimed to evaluate the impacts of atorvastatin on hematoma resolution, cognitive recovery, surgical conversion risk and safety in this cohort to support clinical conservative management. We conducted a single-center, non-randomized, retrospective study enrolling 67 CSDH patients with cognitive impairment between January 2020 and June 2025, who were assigned to atorvastatin group (routine treatment plus atorvastatin 20 mg nightly) or control group (routine treatment alone). Given the modest sample size and retrospective design, conclusions should be interpreted as exploratory. Primary outcomes included 6-month hematoma absorption rate, absorption grade, corrected MoCA score improvement and cognitive impairment incidence; secondary outcomes included serial hematoma volume, MoCA scores, surgical conversion rate and adverse events. Assessments were performed with the assessors blinded to the treatment group allocation. Baseline characteristics were comparable between the atorvastatin group (n = 33) and control group (n = 34). Atorvastatin significantly reduced hematoma volume and elevated MoCA scores at 1, 3 and 6 months, with higher hematoma absorption rate, lower cognitive impairment rate (6.1% vs. 26.5%) and lower surgical conversion rate (6.1% vs. 26.5%) (all P < 0.05). Hematoma absorption was moderate positively correlated with MoCA improvement (*r*_*s*_ = 0.495, P < 0.001),suggesting a potential link, and atorvastatin was an independent protective factor for cognitive recovery (β = 0.980, P < 0.001). No severe adverse events occurred in either group, and mild adverse events were comparable, reversible and manageable. In this exploratory retrospective analysis, atorvastatin 20 mg/day significantly accelerates hematoma absorption, reduces surgical conversion and independently improves cognitive function in CSDH patients with cognitive impairment, with favorable safety. These hypothesis-generating results support its potential as a promising conservative treatment option, warranting validation in prospective trials.

## 1 Introduction

Chronic subdural hematoma (CSDH) is one of the most common neurosurgical conditions, with a striking predilection for the elderly population (Dziho et al., 2025; Read and Edlmann, 2025). With the accelerating aging of the global population and the widespread clinical use of antithrombotic agents, the incidence of CSDH has risen progressively in recent years (Gaist et al., 2017; Arunachalam Sakthiyendran et al., 2025; Cao and Li, 2025). CSDH is characterized by an insidious onset and non-specific clinical manifestations, with classic symptoms including headache, limb hemiplegia, gait instability, and cognitive decline (Kolias et al., 2014; Brennan et al., 2017). Among these, cognitive impairment is the most underrecognized yet most detrimental complication, with an incidence ranging from 50% to 75% (Blaauw et al., 2021; 2023). It directly leads to impaired activities of daily living, prolonged hospital stays, suboptimal rehabilitation outcomes, and even an increased risk of mortality in affected patients (Blaauw et al., 2021; Hennessy et al., 2026), thereby presenting a notable public health challenge (Shibahashi, 2024).

The pathophysiological mechanisms underlying CSDH are complex and multifactorial; the well-recognized core processes include recurrent exudation from immature neovascularization within the hematoma capsule, local chronic inflammatory responses, an imbalance in the fibrinolytic and coagulation systems, and persistent cerebral tissue compression induced by the space-occupying effect of the hematoma (Edlmann et al., 2017; Bounajem et al., 2021; Sağıroğlu et al., 2024; Shimohigoshi et al., 2026). Long-term hematoma compression results in reduced cerebral cortical perfusion, disruption of cerebral structural connectivity, and impairment of synaptic function, thereby inducing or exacerbating cognitive deficits (Machulda and Haut, 2000; Inao et al., 2001). In turn, cognitive impairment diminishes treatment adherence, increases the likelihood of hematoma enlargement, recurrence, and subsequent surgical intervention, forming a vicious cycle that worsens patient outcomes (Moffatt et al., 2019). Therefore, the simultaneous achievement of neuroprotection and cognitive function improvement, while controlling hematoma progression and promoting absorption, has become the core objective of individualized CSDH management (Kolias et al., 2014).

Currently, surgical treatment is the mainstay for patients with obvious discomfort symptoms or significant mass effects (Kim and Lee, 2023; Ma et al., 2023; Chen et al., 2024; Foppen et al., 2024). However, postoperative hematoma recurrence and deterioration of neurological function are still urgent clinical problems that need to be solved (Yang et al., 2024; Bissolo et al., 2026). Consequently, most patients who receive surgical treatment also receive drug therapy (Liu et al., 2025). For patients with small hematoma volumes, no evident risk of cerebral herniation, and mild clinical symptoms, conservative treatment is often adopted, there are few evidence-based guidelines for management (Kan et al., 2024). Moreover, traditional conservative management is limited to supportive measures including bed rest, fluid replacement, and symptomatic pain relief, lacking targeted pharmaceutical interventions that can actively promote hematoma absorption, inhibit inflammatory responses, and preserve neurological function (Liu et al., 2024; Verma et al., 2024). This results in limited overall efficacy, a prolonged hematoma absorption cycle, a higher rate of serious adverse events, and the failure to effectively reverse cognitive decline in affected patients (Miah et al., 2023; Hutchinson et al., 2024).

As a 3-hydroxy-3-methylglutaryl coenzyme A (HMG-CoA) reductase inhibitor, atorvastatin exerts pleiotropic biological effects in the neurological domain beyond its classic lipid-lowering property, which have been extensively validated in preclinical and clinical studies (Morofuji et al., 2022; Aldossary et al., 2024; Yan et al., 2026). Basic research has demonstrated that atorvastatin can inhibit the release of local proinflammatory cytokines within the hematoma, stabilize the vascular endothelial barrier, promote the maturation of immature neovascularization, and reduce capsular exudation (Yang et al., 2022; Kim et al., 2024; Yan et al., 2026). Furthermore, it exerts direct neuroprotective effects through anti-inflammatory, antioxidant, and anti-apoptotic pathways.

In addition to facilitating hematoma absorption, atorvastatin may also directly improve CSDH-related cognitive impairment through pleiotropic neuroprotective mechanisms. These effects likely involve three interrelated pathways: First, by inhibiting the isoprenylation of small GTP-binding proteins (e.g., Rho, Rac), it significantly attenuates chronic neuroinflammation around the hematoma, thereby creating a favorable microenvironment for neuronal recovery (Liao and Laufs, 2005). Second, it selectively upregulates cerebral vascular endothelial nitric oxide synthase (eNOS), thereby improving local cerebral perfusion compromised by hematoma compression (Endres et al., 1998). Most importantly, preclinical studies indicate that atorvastatin exerts direct neurorestorative effects, promoting the expression of synaptic proteins (e.g., synaptophysin), angiogenesis, and the activation of associated neurotrophic signaling pathways, which collectively support the plasticity and reorganization of impaired neural networks (Chen et al., 2003). Therefore, in patients with CSDH, atorvastatin may enhance cognitive recovery not only by alleviating mass effect but also by delivering direct pharmacological neuroprotection.

Clinically, accumulating evidence supports the role of atorvastatin in promoting CSDH hematoma absorption. Several randomized and observational studies have shown that atorvastatin can accelerate hematoma resolution and reduce the need for surgical intervention (Jiang et al., 2018; Wang et al., 2021; Yu et al., 2022). However, However, the clinical evidence for its cognitive benefits remains notably underdeveloped. First, most prior studies primarily focused on radiological and surgical outcomes, with cognitive function assessed only as a secondary or exploratory endpoint, often using brief screening tools (e.g., MMSE) without standardized longitudinal follow-up. Second, and more critically, the high-risk subgroup of CSDH patients with established cognitive impairment at baseline has rarely been the specific focus of pharmacological trials. Consequently, whether the promising hematoma-absorbing effect of atorvastatin translates into tangible, independent cognitive recovery in patients who are already cognitively impaired is unknown. In the present study, cognitive function was assessed using the Montreal Cognitive Assessment (MoCA). The MoCA was selected over the Mini-Mental State Examination (MMSE) due to its superior sensitivity in detecting deficits in executive functions and frontal-subcortical networks, which are commonly impaired in patients with CSDH.

Therefore, a clear dissociation exists between the established evidence for hematoma absorption and the scarce evidence for cognitive improvement. To date, clinical evidence regarding whether atorvastatin can simultaneously and independently ameliorate CSDH-related cognitive impairment while promoting hematoma absorption, as well as its efficacy magnitude and safety profile in this specific population, remains scarce. Based on this knowledge gap, the present study retrospectively analyzed the effects of atorvastatin on hematoma absorption, cognitive function, surgical conversion, and safety specifically in CSDH patients with cognitive impairment, aiming to provide clinical evidence for optimizing conservative treatment strategies and improving long-term prognosis in this cohort.

## 2 Materials and methods

### 2.1 Study design

This was a single-center, retrospective, observational clinical study with per-protocol (PP) set analysis. The analysis utilized data from a standardized institutional clinical pathway for the conservative management of CSDH patients, which mandated a structured schedule for imaging, cognitive assessment, and rehabilitation. All research procedures were conducted in accordance with the ethical principles of the Declaration of Helsinki (2013 revision) and were approved by the Ethics Committee of West China Hospital of Sichuan University-Ziyang Hospital (Approval No. 2024199). Written informed consent from patients was waived by the Ethics Committee due to the exclusive use of de-identified retrospective clinical data.

### 2.2 Study subjects

Patients who were diagnosed with CSDH via cranial computed tomography (CT) or magnetic resonance imaging (MRI) and complicated by cognitive impairment at the Department of Neurosurgery, West China Hospital of Sichuan University-Ziyang Hospital, from January 2020 to June 2025 were enrolled as study participants.

#### 2.2.1 Inclusion criteria

① Aged ≥18 years; ② Confirmed diagnosis of CSDH based on cranial CT/MRI findings in accordance with clinical diagnostic criteria (Kan et al., 2024); ③ No emergency surgical indications: baseline hematoma volume ≤50 mL (measured by the Tada formula), maximum thickness of the hematoma ≤2 cm, maximum midline shift ≤0.5 cm; ④ Conscious with a Glasgow Coma Scale (GCS) score ≥13 points and able to cooperate with MoCA scale assessment; ⑤ Complicated by CSDH-related cognitive impairment: corrected MoCA score <26 points (1-point correction for patients with ≤12 years of education), normal cognitive function 6 months prior to diagnosis, and no history of primary dementia; ⑥ Completed 6 months of standardized follow-up with complete clinical, imaging, and cognitive assessment data; ⑦ Patients in the atorvastatin group received atorvastatin for ≥3 months with a medication compliance rate ≥80%.

#### 2.2.2 Exclusion criteria

① Acute or subacute subdural hematoma, intracranial tumor, hydrocephalus, cerebral contusion, or other intracranial pathological lesions; ② Complicated by severe cardio-cerebrovascular diseases (e.g., heart failure, unstable angina pectoris, myocardial infarction within 3 months), malignant tumors, coagulation dysfunction, or severe organ failure of the liver, kidney, or other vital organs; ③ A history of pre-existing cognitive impairment, Alzheimer’s disease, vascular dementia, Parkinson’s disease, or other neurodegenerative diseases; ④ Indistinct imaging data or missing key indicators (e.g., hematoma volume, MoCA score) precluding data extraction; ⑤ Hypersensitivity to atorvastatin or other statins, or presence of medication contraindications (e.g., active liver disease, persistent transaminase elevation, history of myopathy); ⑥ Receipt of corticosteroid therapy or drugs affecting coagulation or cognitive function within 1 month before enrollment or during the study period; ⑦ Incomplete follow-up data, loss to follow-up, or violation of the study protocol.

#### 2.2.3 Treatment allocation and potential for allocation bias

This was a non-randomized, retrospective study. The assignment to the atorvastatin group or control group was based on real-world clinical decisions made by treating physicians at the time of patient management, not by a pre-specified randomization protocol. The decision to add atorvastatin to routine care was primarily influenced by a combination of the following factors: 1) the patient’s baseline lipid profile (patients with elevated low-density lipoprotein cholesterol [LDL-C] levels, typically ≥2.6 mmol/L, were more likely to be prescribed atorvastatin for cardiovascular risk management in accordance with contemporaneous guidelines); 2) the clinician’s individual judgment regarding the perceived benefit of adjuvant pharmacotherapy; and 3) patient preference and economic considerations. It is important to note that this allocation was not primarily driven by baseline disease severity, as confirmed by the comparable baseline characteristics between the two groups (see Results, Table 1), including hematoma volume, midline shift, MoCA scores, and comorbidity profiles (all P > 0.05). Nevertheless, we acknowledge that unmeasured confounding factors (e.g., subtle differences in inflammatory status, genetic predisposition, or unrecorded lifestyle factors) and indication bias could persist. To mitigate the impact of these potential biases on outcome estimation, we performed rigorous multivariate regression analyses adjusting for key baseline covariates (see Statistical Analysis) and conducted sensitivity analyses (see Statistical Analysis and Results).

**TABLE 1:** Clinical and demographic data in the two study groups.

Parameter	Atorvastatin group (n = 33)	Control group (n = 34)	Test statistic	*p*-value
Sex (male,%)	26 (78.8%)	26 (76.5%)	χ^2^ = 0.052	0.820
Age (year)	67.33 ± 7.53	67.03 ± 8.79	t = 0.152	0.880
Years of education (year)	6.21 ± 3.07	6.29 ± 2.82	t = 0.114	0.910
Smoking history (yes)	16 (48.5%)	13 (38.2%)	χ^2^ = 0.717	0.464
Alcohol history (yes)	16 (48.5%)	15 (44.1%)	χ^2^ = 0.128	0.808
Hypertension (yes)	10 (30.3%)	13 (38.2%)	χ^2^ = 0.467	0.609
Diabetes (yes)	6 (18.2%)	8 (23.5%)	χ^2^ = 0.290	0.765
Trauma history (yes)	19 (57.6%)	18 (52.9%)	χ^2^ = 0.145	0.807
Baseline ALT (U/L)	21.73 ± 7.98	23.94 ± 8.70	t = 1.085	0.282
Baseline CK (U/L)	87.64 ± 25.15	85.79 ± 21.60	t = 0.322	0.749
Baseline MoCA score	22.21 ± 1.65	22.68 ± 1.51	t = 1.200	0.234
Baseline hematoma volume (mL)	27.67 ± 6.07	27.41 ± 4.79	t = 0.191	0.849

### 2.3 Sample size and grouping

**FIGURE 1:**
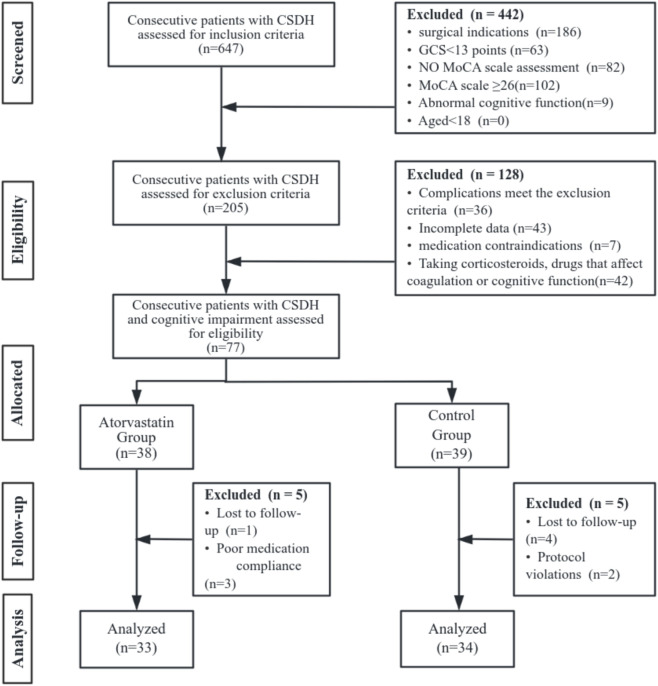
Study flow diagram.

A total of 77 patients were initially enrolled in the study and non-randomly assigned to the atorvastatin group (38 cases) or the control group (39 cases) based on actual clinical therapeutic regimens. All statistical analyses were performed on the PP set. A total of 10 patients were excluded from the PP set during the study period, including 5 cases lost to follow-up, 3 cases with poor medication compliance, and 2 cases with protocol violations. Finally, 67 patients were included in the PP set (33 in the atorvastatin group and 34 in the control group), all of whom completed the 6-month full-course follow-up and data collection, as shown by the study flow diagram in Figure 1.

### 2.4 Therapeutic regimens

#### 2.4.1 Control group

Patients received routine conservative treatment for CSDH combined with standardized cognitive rehabilitation occupational therapy. Specific measures included: bed rest with the head of the bed elevated at 15°–30° to avoid strenuous exercise; antihypertensive therapy with amlodipine, valsartan, or other agents to maintain blood pressure at 100–140/60–90 mmHg in hypertensive patients; antidiabetic therapy with oral hypoglycemic agents or insulin to control fasting blood glucose <7.0 mmol/L and 2-h postprandial blood glucose <10.0 mmol/L in diabetic patients; and oral ibuprofen sustained-release capsules 0.3 g as needed for patients with severe headache (no more than twice daily, cumulative use ≤7 days). Cognitive rehabilitation training was individually tailored by therapists based on the assessment results, administered at a frequency of five sessions per week (30 min per session) for a total duration of 24 weeks or until cognitive function returned to normal levels (MoCA ≥26), whichever occurred first.

#### 2.4.2 Atorvastatin group

On the basis of the routine treatment administered to the control group, atorvastatin calcium tablets (Pfizer Pharmaceuticals, 20 mg/tablet; National Medicine Approval No. H20051408) were initiated within 48 h after enrollment at a dose of 20 mg once daily, taken orally at bedtime, with a total treatment course of 6 months. Liver function (alanine transaminase, ALT) and creatine kinase (CK) levels were monitored regularly during treatment: treatment was discontinued immediately and symptomatic management initiated if transaminase levels increased by more than 3 times the upper limit of normal (ULN) or CK levels exceeded 10 times the ULN; the dose was reduced to 10 mg daily for continuous observation if transaminase levels increased by 1.5–3 times the ULN, with a recheck 2 weeks later, and treatment was discontinued if transaminase levels remained >1.5 times the ULN.

#### 2.4.3 Standardization of care under the clinical pathway

All enrolled patients were managed according to the aforementioned standardized clinical pathway. This pathway mandated: 1) scheduled cranial CT imaging at 1, 3, and 6 months, 2) serial cognitive assessment using alternate MoCA forms at the same intervals, and 3) a structured cognitive rehabilitation program. The rehabilitation program, tailored by the same team of therapists based on initial assessment, consisted of five 30-min sessions per week. The therapy continued for 24 weeks or until the patient’s corrected MoCA score reached ≥26, whichever occurred first. Adherence to rehabilitation and attendance at scheduled follow-ups were monitored as part of routine clinical practice. All data regarding compliance, imaging, and cognitive outcomes presented in this study were retrospectively extracted from these clinical records.

Quality Control:To ensure the comparability of non-pharmacological intervention, adherence to the rehabilitation schedule was strictly monitored. The mean attendance rate was high and comparable between the atorvastatin group (96.2%) and the control group (95.7%) (P = 0.682). To minimize assessment bias, outcome assessment was performed with the assessors blinded to treatment group allocation. Specifically, the radiologists measuring hematoma volumes and the researchers administering the MoCA assessments conducted their evaluations unaware of patient assignment to the atorvastatin or control group. The inter-rater reliability for these assessments was confirmed to be high (Cohen’s Kappa for MoCA = 0.85; ICC for hematoma volume = 0.91), as detailed in Section “Results”.

### 2.5 Data collection and follow-up

#### 2.5.1 Data collection content

Baseline data were retrospectively extracted from the hospital electronic medical record system, including sex, age, years of education, smoking history, alcohol history, hypertension, diabetes, trauma history, baseline MoCA score, and baseline hematoma volume ALT and CK levels. The baseline MoCA assessment was performed after initial clinical stabilization, typically within 48 h of admission, when the patient was alert and cooperative (GCS ≥13).

Follow-up data were collected at 1, 3, and 6 months post-treatment (Kan et al., 2024), including:

##### 2.5.1.1 Imaging indicators

Cranial plain CT scans were performed at each time point, and hematoma volume was independently calculated by two senior radiologists using the Tada formula: Hematoma volume = π/6 × length × width × height (unit: cm). If the measurement difference between the two radiologists exceeded 5%, a third senior radiologist conducted a review, and the review result was regarded as the final value. Hematoma absorption rate was calculated as: (Baseline hematoma volume − 6-month hematoma volume)/Baseline hematoma volume × 100%. Hematoma absorption was classified into three grades: good absorption (>66.6%), partial absorption (33.3%–66.6%), and poor absorption (<33.3%).

##### 2.5.1.2 Cognitive function indicators

MoCA scores were independently assessed by two uniformly trained researchers at each time point. The MoCA scale evaluates seven cognitive domains, including visuospatial and executive function, naming, attention, language, abstract thinking, memory, and orientation, with a total score ranging from 0 to 30 points (Nasreddine et al., 2005). The MoCA has demonstrated good sensitivity in detecting cognitive impairment in various neurological conditions, including CSDH (Blaauw et al., 2023). A 1-point correction was applied for patients with ≤12 years of education. To minimize potential practice effects from repeated testing, alternate equivalent forms of the MoCA (Chinese version) were utilized at different follow-up visits, and the assessment intervals were sufficiently long (≥4 weeks). All assessments were performed in strict accordance with the standardized operation manual.

##### 2.5.1.3 Safety indicators

Liver function, CK levels, and drug-related adverse events (e.g., myalgia, gastrointestinal discomfort, rash).

##### 2.5.1.4 Surgical conversion

The number of patients converted to surgical treatment due to hematoma enlargement (volume >60 mL), midline shift >0.5 cm, or a GCS score decrease of ≥2 points.

#### 2.5.2 Follow-up protocol

A combined approach of outpatient follow-up and telephone follow-up was adopted to complete the 6-month follow-up, with the following key time points: ① Enrollment: Collection of baseline data, cranial CT, and MoCA score; ② 1 month: Cranial CT, MoCA score, medication compliance, and adverse events assessment; ③ 3 months: Cranial CT, MoCA score, and complication recording; ④ 6 months: Cranial CT, MoCA score, prognosis evaluation, and adverse event documentation. Loss to follow-up was defined as failure to attend scheduled follow-up, ineffective contact after three telephone calls, loss of contact for more than 4 weeks, or voluntary withdrawal from the study.

All scheduled follow-up visits for the assessment of primary outcomes—including cranial CT scans and MoCA evaluations at 1, 3, and 6 months—were conducted during in-person outpatient clinic visits to ensure standardization. Telephone contact was reserved for appointment reminders, checking medication adherence between visits, and initial screening of reported adverse events; any potential adverse event identified via telephone was subsequently verified and documented at the next in-person visit.

#### 2.5.3 Data quality control

MoCA score assessment and hematoma volume measurement were independently completed by two researchers under mutual single-blinding. Cohen’s Kappa test was used to evaluate the consistency of MoCA scores (Kappa >0.6 indicating good consistency), and the intraclass correlation coefficient (ICC) was used to assess the consistency of hematoma volume measurements (ICC >0.75 indicating good consistency), ensuring the reliability and stability of all collected data.

### 2.6 Definition of outcome measures

#### 2.6.1 Primary outcome measures

① 6-month hematoma absorption rate and absorption grade; ② 6-month incidence of cognitive impairment (corrected MoCA score <26 points); ③ 6-month corrected MoCA score and MoCA score improvement value (**Δ**MoCA = 6-month MoCA score − baseline MoCA score).

#### 2.6.2 Secondary outcome measures

① Hematoma volume and corrected MoCA score at each follow-up time point; ② 6-month surgical conversion rate; ③ 6-month incidence of adverse events (drug-related adverse events, rebleeding, infection, death).

### 2.7 Statistical analysis

All statistical analyses were performed using IBM SPSS Statistics 22.0 software, with all analyses based on the PP set. A two-tailed test was used, with a significance level of α = 0.05. Exact P-values was reported, and a *P*-value <0.05 was considered statistically significant. The selection of covariates for multivariate regression was based on *a priori* clinical rationale and univariate analysis results (P < 0.10). The independent effect of atorvastatin was analyzed using multivariate regression, with 6-month hematoma absorption rate, MoCA score improvement value, and surgical conversion rate as the dependent variables, adjusted for years of education, baseline MoCA score, baseline hematoma volume, and alcohol consumption.① Continuous variables: Normally distributed data were expressed as mean ± standard deviation, and non-normally distributed data as median (interquartile range, IQR). Categorical variables were expressed as n (%). ② Baseline balance test: An independent samples *t*-test was used for normally distributed continuous variables, the Mann-Whitney *U* test for non-normally distributed continuous variables, and the χ^2^ test or Fisher’s exact probability method for categorical variables. ③ Repeated measures analysis of variance: Used to evaluate the time effect, intergroup effect, and time × group interaction effect of hematoma volume and MoCA scores; the Greenhouse-Geisser correction was applied when the sphericity assumption was violated, and the Bonferroni method was used for *post hoc* intra-group comparisons. ④ Correlation analysis: Spearman’s rank correlation analysis was used to investigate the correlation between hematoma absorption rate and MoCA score improvement value, as the MoCA score is an ordinal clinical scale. ⑤ Multiple linear regression analysis: The 6-month MoCA score improvement value (ΔMoCA) was set as the dependent variable. Variables that showed a potential association in the univariate analyses (P < 0.10), namely, baseline MoCA score and atorvastatin treatment, were entered simultaneously into a multivariable linear regression model using the ‘Enter’ method. This approach, based on both statistical and clinical rationale, allows for the assessment of the independent contribution of these pre-specified factors while maintaining model stability given the sample size. Model diagnostics were performed to ensure validity. Collinearity was assessed using the variance inflation factor (VIF <10 was considered acceptable) and tolerance (tolerance >0.1), and no substantial multicollinearity was detected. The assumptions of normality, homoscedasticity, and linearity were evaluated by visual inspection of the residual plots (Q-Q plot, scale-location plot, and residual vs. fitted plot, respectively), and no major violations were observed.⑥ Surgical conversion rate and adverse events rate: Analyzed using the χ2 test or Fisher’s exact probability method.⑦ Sensitivity Analyses: To assess the robustness of the primary findings against potential confounding, sensitivity analyses were performed. For the continuous outcomes (ΔMoCA and hematoma absorption rate), the non-parametric Mann-Whitney U test was applied to the residuals obtained from an analysis of covariance (ANCOVA) model, which adjusted for the following covariates: sex, age, smoking, alcohol use, hypertension, diabetes, trauma history, baseline MoCA score, and baseline hematoma volume. For the binary outcome of surgical conversion, a multivariable logistic regression model adjusted for the same set of covariates was used to calculate the adjusted odds ratio (OR) and 95% confidence interval (CI).

## 3 Results

### 3.1 Consistency test of data collection

Consistency testing of MoCA score assessment and hematoma volume measurement by two independent researchers showed a Cohen’s Kappa coefficient of 0.85 for MoCA scores, indicating good consistency in cognitive assessment; the ICC for hematoma volume measurement was 0.91, indicating excellent consistency in imaging measurement. The reliability and stability of data collection in this study met the rigorous requirements of clinical research.

### 3.2 Comparison of baseline characteristics between the two groups

No statistically significant differences were observed in baseline characteristics between the atorvastatin group and the control group, including sex, age, years of education, smoking history, alcohol history, hypertension, diabetes, trauma history, baseline ALT, baseline CK, baseline MoCA score, and baseline hematoma volume (all *P* > 0.05). These results indicated that the two groups were well-balanced and comparable, with no significant selection bias and good intergroup comparability (Table 1).

Multivariate regression analysis showed that atorvastatin treatment was an independent influencing factor for 6-month hematoma absorption rate, MoCA score improvement value, and surgical conversion rate, after adjustment for years of education, baseline MoCA score, baseline hematoma volume, and alcohol consumption. Detailed results were presented in Table 2.

**TABLE 2:** Independent effects of atorvastatin on outcome measures by multivariate regression analysis.

Outcome measures	Factor	Estimate	P-Value	95% CI
Hematoma absorption rate	Atorvastatin treatment	B = 17.790	<0.001	12.049~23.531
MoCA score improvement value		B = 0.987	<0.001	0.561~1.412
Surgical conversion rate		OR = 0.188	0.045	0.037~0.962

### 3.3 Comparison of hematoma volume and hematoma absorption rate at each follow-up time point

#### 3.3.1 Hematoma volume and its changes (ΔHematoma volume) at each time point

Repeated measures analysis of variance revealed significant time main effects (*F* = 289.786, *P* < 0.001), intergroup main effects (*F* = 13.700, *P* < 0.001), and time × group interaction effects (*F* = 6.780, *P* = 0.001) for hematoma volume. These findings suggested that hematoma volume decreased significantly with the extension of treatment time in both groups, and the atorvastatin group exhibited a significantly greater reduction in hematoma volume compared with the control group. Intergroup comparison of changes (Δhematoma volume) (baseline vs. post-treatment) at each single time point showed that the reduction in hematoma volume in the atorvastatin group was significantly greater than that in the control group at 1, 3, and 6 months post-treatment (all *P* < 0.05). Intra-group comparisons demonstrated that hematoma volume at all follow-up time points was significantly lower than the baseline in both groups (all *P* < 0.001) (Table 3).

**TABLE 3:** Comparison of hematoma volume and its changes (ΔHematoma volume) between two groups at various time points (ml).

Time point	Hematoma volume		ΔHematoma volume			
	Atorvastatin group (n = 33)	Control group (n = 34)	Atorvastatin group (n = 33)	Control group (n = 34)	t-value	p-value
Baseline	27.667 ± 6.066	27.412 ± 4.787	​	​	t = 0.191	0.849
1 Month	28.061 ± 9.526	32.471 ± 9.173	−0.39 ± 8.941	−5.06 ± 9.048	t = 2.122	0.038
3 Months	16.303 ± 4.740	22.618 ± 5.411	11.36 ± 5.754	4.79 ± 5.912	t = 4.607	<0.001
6 Months	9.485 ± 3.572	13.676 ± 4.353	18.18 ± 5.046	13.74 ± 5.130	t = 3.575	0.001

#### 3.3.2 6-Month hematoma absorption rate and absorption grade

The 6-month hematoma absorption rate in the atorvastatin group was significantly higher than that in the control group (*t* = 5.063, *P* < 0.001). There was a statistically significant difference in the distribution of hematoma absorption grades between the two groups (χ^2^ = 10.633, *P* = 0.005). Pairwise comparisons showed significant differences between the good absorption group and the partial absorption group, as well as between the good absorption group and the poor absorption group (all *P* < 0.05), while no significant difference was observed between the partial absorption group and the poor absorption group (*P* > 0.05). These results indicated that the intergroup difference in hematoma absorption was mainly reflected in the proportion of patients with good hematoma absorption (Table 4).

**TABLE 4:** Comparison of hematoma absorption rate and grade between two groups of patients at 6 months.

Parameter	Atorvastatin group (n = 33)	Control group (n = 34)	Test statistic	p-value
Hematoma absorption rate (%)	65.626 ± 10.438	49.489 ± 15.146	t = 5.063	<0.001
Hematoma absorption grade	​	​	χ^2^ = 10.633	0.005
Good absorption (A)	16 (48.5%)	5 (14.7%)	​	​
Partial absorption (B)	17 (51.5%)	26 (76.5%)	​	​
Poor absorption (C)	0 (0%)	3 (8.8%)	​	​
A:B	​	​	χ^2^ = 7.591	0.006
A:C	​	​	Fisher	0.028
B:C	​	​	Fisher	0.286

### 3.4 Comparison of MoCA score and cognitive function outcomes at each follow-up time point

#### 3.4.1 MoCA score and its changes (ΔMoCA score) at each time point

Repeated measures analysis of variance revealed significant time main effects (*F* = 808.931, *P* < 0.001), intergroup main effects (*F* = 61.903, *P* < 0.001), and time × group interaction effects (*F* = 26.358, *P* < 0.001) for MoCA score. These results suggested that cognitive function improved significantly with the extension of treatment time in both groups, and the atorvastatin group exhibited a significantly more pronounced elevation in MoCA score compared with the control group. Intergroup comparison of ΔMoCA score (baseline vs. post-treatment) at each single time point showed that the increase in MoCA scores in the atorvastatin group was significantly greater than that in the control group at 1, 3, and 6 months post-treatment (all *P* < 0.05). Intra-group comparisons demonstrated that MoCA score at all follow-up time points were significantly higher than the baseline in both groups (all *P* < 0.001) (Table 5).

**TABLE 5:** Comparison of MoCA score and its changes (ΔMoCA score) between two groups at various time points.

Time point	MoCA score		ΔMoCA score			
	Atorvastatin group (n = 33)	Control group (n = 34)	Atorvastatin group (n = 33)	Control group (n = 34)	t-value	p-value
Baseline	22.21 ± 1.65	22.68 ± 1.51	​	​	t = 1.200	0.234
1 Month	23.21 ± 1.52	23.12 ± 1.57	1.00 ± 0.661	0.44 ± 0.660	t = 3.461	0.001
3 Months	25.91 ± 1.51	24.35 ± 1.63	3.70 ± 0.770	1.68 ± 0.843	t = 10.236	<0.001
6 Months	27.58 ± 1.41	26.97 ± 1.64	5.36 ± 0.822	4.29 ± 0.970	t = 4.861	<0.001

#### 3.4.2 6-Month cognitive function status

Using a corrected MoCA score <26 points as the diagnostic criterion for cognitive impairment, the 6-month incidence of cognitive impairment was 6.1% (2/33) in the atorvastatin group and 26.5% (9/34) in the control group, with a statistically significant difference between the two groups (χ^2^ = 5.084, *P* = 0.045).

### 3.5 Correlation between hematoma absorption rate and the MoCA score improvement value

Spearman’s correlation analysis showed that the 6-month hematoma absorption rate was moderate positively correlated with the MoCA score improvement value (*r*_*s*_ = 0.495, *P* < 0.001), suggesting that a higher degree of hematoma absorption was associated with a more significant improvement in cognitive function in CSDH patients.

### 3.6 Multivariate linear regression analysis of 6-month cognitive function improvement

#### 3.6.1 Univariate analysis

With the 6-month MoCA score improvement value as the dependent variable, univariate analysis (independent samples t-test or Pearson correlation analysis was used as appropriate) was performed for variables including sex, age, years of education, smoking history, alcohol history, hypertension, diabetes, trauma history, baseline MoCA score, baseline hematoma volume, surgical conversion rate, and atorvastatin treatment. The results identified baseline MoCA score and atorvastatin treatment as factors associated with the 6-month cognitive function improvement (all *P* < 0.05), which were subsequently included in the multivariate linear regression analysis (Table 6).

**TABLE 6:** Univariate analysis of factors associated with 6-month cognitive function improvement.

Variables	r-Value	Mean ± standard	t-Value	P-value	95% CI
Sex (M/F)	​	4.83 ± 1.024/4.80 ± 1.146	0.087	0.931	−0.588~0.642
Age (year)	−0.093	​	​	0.456	​
Years of education (year)	0.035	​	​	0.779	​
Smoking history (yes/No)	​	4.83 ± 1.071/4.82 ± 1.036	0.046	0.964	−0.509~0.533
Alcohol history (yes/No)	​	4.77 ± 0.956/4.86 ± 1.125	0.338	0.737	−0.601~0.427
Hypertension (yes/No)	​	4.87 ± 1.058/4.80 ± 1.047	0.274	0.785	−0.466~0.614
Diabetes (yes)	​	4.57 ± 0.938/4.89 ± 1.068	1.006	0.318	−0.941~0.311
Trauma history (Yes)	​	4.78 ± 1.031/4.87 ± 1.074	0.321	0.749	−0.598~0.433
Baseline hematoma volume (mL)	−0.130	​	​	0.294	​
Surgical conversion rate (Yes/No)	​	4.55 ± 0.688/4.88 ± 1.096	0.957	0.342	−1.017~0.358
Baseline MoCA score	0.362	​	​	0.003	​
Atorvastatin treatment (yes/No)	​	5.36 ± 0.822/4.29 ± 0.970	4.861	<0.001	−1.509 ∼ −0.630

#### 3.6.2 Multivariate linear regression analysis

Multivariate linear regression analysis was performed with the 6-month MoCA score improvement value as the dependent variable and the variables screened by univariate analysis as independent variables. The regression model was statistically significant (*R* ^2^ = 0.350, adjusted *R* ^2^ = 0.330), indicating that the independent variables could explain approximately 33.0% of the variance in the dependent variable. Atorvastatin treatment was identified as an independent protective factor for cognitive function improvement in CSDH patients (β = 0.980, P < 0.001, 95%CI: 0.559–1.402), which further verified the pharmacological specificity of atorvastatin’s neuroprotective effect—this effect is not a secondary consequence of hematoma absorption merely, but a direct regulatory effect on the neurobiological processes of cognitive impairment. Baseline MoCA score was an independent prognostic factor for cognitive function improvement (β = 0.192, *P* = 0.006, 95%CI: 0.058–0.326), indicating that a higher baseline MoCA score was associated with a more pronounced improvement in cognitive function at 6 months, whereas a lower baseline MoCA score predicted a poorer cognitive recovery (Table 7).

**TABLE 7:** Multivariate linear regression analysis of factors associated with 6-month cognitive function improvement.

Variables	B	Se	t	P-Value	95% CI
Atorvastatin treatment (yes)	0.980	0.211	4.646	<0.001	0.559~1.402
Baseline MoCA score	0.192	0.067	2.872	0.006	0.058~0.326
Constant	7.671	1.583	4.847	<0.001	4.059~10.832

### 3.7 Comparison of surgical conversion rate and adverse events rate between the two groups

#### 3.7.1 Surgical conversion rate

Within 6 months of treatment, 2 patients in the atorvastatin group were converted to surgical treatment due to hematoma enlargement and aggravated clinical symptoms, resulting in a surgical conversion rate of 6.1%. In contrast, 9 patients in the control group underwent surgical intervention, with a surgical conversion rate of 26.5%. The surgical conversion rate in the atorvastatin group was significantly lower than that in the control group (χ^2^ = 5.084, *P* = 0.024).

#### 3.7.2 Adverse events rate

No serious adverse events (e.g., death, hemiplegia, vital organ insufficiency) were reported in either group during the treatment period. Four patients in the atorvastatin group experienced mild drug-related adverse events, including 3 cases of mild transaminase elevation and 1 case of mild myalgia, resulting in an adverse events rate of 11.8%. No study-related adverse events were observed in the control group. Fisher’s exact probability method showed no statistically significant difference in the incidence of adverse events between the two groups (*P* = 0.114). All patients with mild adverse events achieved complete resolution after symptomatic treatment or dose adjustment, and no patients discontinued treatment due to adverse events.

### 3.8 Sensitivity analyses

The results of the sensitivity analyses supported the primary findings. After adjustment for the specified covariates, the atorvastatin group maintained a significantly greater improvement in MoCA score (Z = 4.293, P < 0.001) and a higher hematoma absorption rate (Z = 4.585, P < 0.001) compared to the control group. Furthermore, in the adjusted logistic regression model, atorvastatin treatment remained independently associated with a reduced risk of surgical conversion (adjusted OR = 0.18, 95% CI: 0.034–0.995, P = 0.049).

## 4 Discussion

This study is the first retrospective clinical drug study to focus on the dual effects of atorvastatin calcium in promoting hematoma absorption and ameliorating cognitive impairment in CSDH patients with concomitant cognitive dysfunction. The results confirmed that atorvastatin calcium can simultaneously achieve three key clinical benefits: accelerating hematoma absorption, improving cognitive function, and reducing the risk of surgical conversion, with a favorable safety profile. These findings provide crucial evidence-based support for the non-surgical management of these patients, particularly through pharmacological interventions.

First, the present study confirmed that atorvastatin can significantly accelerate CSDH hematoma absorption and reduce the surgical conversion rate, which is highly consistent with the conclusions of previous clinical studies (Jiang et al., 2018; Wang et al., 2021; Liu et al., 2025). The progressive development of CSDH is driven by recurrent exudation from immature blood vessels in the hematoma capsule, chronic inflammatory infiltration, and hyperfibrinolysis in the local microenvironment. The observed acceleration of hematoma absorption in our atorvastatin group can be plausibly explained by its well-documented pleiotropic effects, which target these core pathophysiological processes: reduction of vascular inflammation and stabilization of the neovasculature within the hematoma capsule, leading to decreased leakage. This pathophysiological intervention is consistent with the faster hematoma volume reduction and higher resolution rate observed in our atorvastatin group.

Second, the most clinically innovative finding of this study is that atorvastatin exerts an independent effect in ameliorating CSDH-related cognitive impairment, as shown in multivariate regression. CSDH-related cognitive impairment is multifactorial, involving cerebral compression, reduced perfusion, and neuroinflammation. Atorvastatin’s beneficial effect on cognition may operate through a dual mechanism: indirectly, by alleviating mass effect and improving perfusion through hematoma resolution (Xu et al., 2017; Xu et al., 2023); and directly, through its intrinsic neuroprotective properties (Mounier et al., 2021). The independent association found in our regression model suggests a direct pharmacological effect beyond mere hematoma removal. This is supported by the drug’s known capacities to mitigate neuroinflammation and oxidative stress, and to promote endothelial function and cerebral blood flow, all of which are pathways implicated in cognitive preservation (Mounier et al., 2021; Yuan et al., 2024; Attiq et al., 2025). Our finding that atorvastatin treatment was an independent protective factor for cognitive recovery aligns with this broader neuroprotective profile.

Third, the present study found a moderate positive correlation (*r*_*s*_ = 0.495) between hematoma absorption rate and MoCA score improvement. This association suggests a potential link, but does not establish causality. It is plausible that relieving cerebral tissue compression through hematoma absorption contributes to cognitive recovery, while the direct neuroprotective effect of atorvastatin, as indicated by its independent association in regression analysis, provides an additional therapeutic avenue. Therefore, these findings should be interpreted as hypothesis-generating. They raise the possibility that for CSDH patients with cognitive impairment, a therapeutic strategy combining hematoma resolution with direct neuroprotection (e.g., via atorvastatin) may be superior to supportive care alone in breaking the vicious cycle of “hematoma enlargement → cerebral compression → cognitive deterioration”. This hypothesis warrants testing in prospective studies designed to delineate the specific contributions of mechanical decompression versus pharmacological neuroprotection to cognitive outcomes.

Fourth, the robustness of our primary findings is further reinforced by the results of the comprehensive sensitivity analyses. After adjusting for a panel of clinically relevant baseline covariates, the significant benefits of atorvastatin on both cognitive improvement (ΔMoCA) and hematoma absorption rate persisted with high statistical significance (both P < 0.001). This indicates that the observed therapeutic effects are unlikely to be solely attributable to imbalances in the measured patient characteristics at baseline. Importantly, the reduced risk of surgical conversion associated with atorvastatin also remained evident in the adjusted model (adjusted OR = 0.18, P = 0.049), albeit with a P-value at the conventional significance threshold. The consistency of the direction and magnitude of effect across all sensitivity analyses—spanning continuous neurological, radiological, and critical binary clinical endpoints—strengthens the internal validity of our conclusions. It suggests that the pleiotropic benefits of atorvastatin in this patient cohort are robust to potential confounding by the factors we were able to measure and adjust for.

In terms of safety, the dosage of 20 mg/d was selected in this study due to its favorable therapeutic safety window, and previous studies have confirmed that this dosage can effectively reduce hematoma volume and improve clinical outcomes with definite efficacy (Jiang et al., 2018; Liu et al., 2025). The present study used a conventional clinical dose of atorvastatin (20 mg/day), and the observed adverse events were limited to transient mild transaminase elevation and mild myalgia. No severe adverse events such as severe liver injury, rhabdomyolysis, or cardiovascular events occurred, which is consistent with the findings of large-scale global statin safety studies. (Bumpetch et al., 2023; Monteiro et al., 2024; Yuan et al., 2025). These findings support the long-term safety and tolerability of this dose in elderly CSDH patients with multiple comorbidities, and provide a reliable dose reference for clinical practice.

Nevertheless, the numerically higher incidence of mild adverse events in the atorvastatin group (11.8% vs. 0%) merits careful consideration. Although all events were mild and reversible, and no severe adverse events occurred, the absolute difference in event rates is clinically non-negligible. This finding underscores the importance of regular monitoring of liver function and creatine kinase levels during atorvastatin therapy in this elderly population. Future prospective studies with larger sample sizes are needed to more precisely characterize the safety profile of atorvastatin in CSDH patients.

Since its efficacy for CSDH was first reported by Chinese researchers, atorvastatin has become the first-line pharmacological treatment for mild CSDH (Jiang et al., 2018). Multiple studies have confirmed its efficacy in promoting hematoma absorption and reducing recurrence risk, with a recommended clinical dose of 20 mg per day and a typical course of 8–12 weeks, showing particular potential for improving prognosis in the very elderly with poor general condition who cannot tolerate surgery (Liu et al., 2025). The recent meta-analyses of clinical trials have confirmed that atorvastatin significantly increases the rate of hematoma absorption and reduces the risk of recurrence in patients with CSDH, providing high-level quantitative evidence for its clinical application (Khan et al., 2024; Monteiro et al., 2024). The Chinese Guidelines for the Diagnosis and Treatment of Chronic Subdural Hematoma (2023 Edition) recommend atorvastatin as an individualized option for asymptomatic patients with small-volume hematomas. Clinicians should note that atorvastatin therapy for CSDH represents an off-label application. Its implementation, therefore, must follow standardized off-label use protocols, which mandate a formal benefit-risk analysis, documented informed consent, and institutional review in accordance with relevant regulations. This structured approach is essential to mitigate potential risks and is particularly crucial when managing patients who may later become candidates for surgical intervention.

This study has several inherent limitations that should be acknowledged. First, it is a single-center, retrospective, non-randomized study, which may be subject to potential selection bias and confounding factors. However, we implemented rigorous statistical adjustments, including multivariate regression analysis adjusted for age, years of education, baseline MoCA score, baseline hematoma volume, trauma history, diabetes, and alcohol consumption, to mitigate these risks and enhance the robustness of the findings. Propensity score matching (PSM) was not performed in this study, as this method would further reduce the effective sample size and lead to insufficient statistical power for reliable inference on core outcomes, especially for surgical conversion with a low event rate. Furthermore, while the primary sensitivity analyses confirmed the robustness of our main findings, more extensive subgroup analyses (e.g., stratified by baseline cognitive severity or hematoma density) were not feasible due to the limited sample size. This precluded a detailed exploration of potential efficacy differences across patient subpopulations. Future well-designed multi-center, randomized controlled trials (RCTs) with a larger sample size are needed to further verify the conclusions of this study. Second, the sample size of the present study is relatively limited, which may affect the extrapolation of the study results to the general population; expanding the sample size in subsequent studies will help to improve the external validity of the findings. Third, the follow-up period of this study is 6 months, and the long-term cognitive prognosis, hematoma recurrence risk, and long-term safety of atorvastatin in CSDH patients remain to be investigated with an extended follow-up period. Fourth, given the retrospective exploratory nature of this clinical study, this study did not explore the underlying molecular mechanisms of atorvastatin in ameliorating CSDH-related cognitive impairment; future basic and translational research is needed to elucidate the specific signaling pathways involved. Fifth, baseline LDL-C data were not available for all patients, preventing adjustment for potential confounding by lipid profiles that may have influenced real-world prescribing decisions. Sixth, the exclusion of patients on anticoagulant or antiplatelet therapy limits the generalizability of our findings to the broader elderly CSDH population, where such medication use is common. Despite these limitations, the present study maximized the control of potential biases through strict inclusion and exclusion criteria, outcome assessment performed by personnel blinded to group allocation, baseline balance matching, and comprehensive statistical corrections, ensuring that the study conclusions have high reliability and clinical guiding value.

In summary, atorvastatin calcium exerts a definite therapeutic effect and a favorable safety profile in CSDH patients with cognitive impairment. It can simultaneously promote hematoma absorption, ameliorate cognitive function, and reduce the risk of surgical conversion, and thus has important clinical application value. Taken together, the findings of this study support the consideration of atorvastatin calcium (20 mg/day) as a promising first-line conservative treatment option for CSDH patients complicated by cognitive impairment, pending validation in prospective, randomized controlled trials.

## 5 Conclusion

1. Atorvastatin calcium (20 mg/day) can significantly accelerate hematoma absorption, increase the hematoma absorption rate, and reduce the surgical conversion rate in patients with chronic subdural hematoma complicated by cognitive impairment.
2. Atorvastatin exerts an independent protective effect in ameliorating cognitive function and reducing the incidence of cognitive impairment in this patient cohort.
3. The degree of hematoma absorption is positively correlated with cognitive function improvement, and atorvastatin exerts dual therapeutic effects of relieving cerebral tissue compression and direct neuroprotection in CSDH patients with cognitive impairment.
4. Atorvastatin demonstrates a favorable safety profile with mild, manageable adverse events in elderly CSDH patients with multiple comorbidities, making it suitable for routine clinical promotion and application.

## Data Availability

The original contributions presented in the study are included in the article/supplementary material, further inquiries can be directed to the corresponding authors.
